# Comparison of Physiological and Biochemical Autonomic Indices in Children with and without Autism Spectrum Disorders

**DOI:** 10.3390/medicina55070346

**Published:** 2019-07-07

**Authors:** Remya Bharath, Shailaja S. Moodithaya, Shrinivasa U. Bhat, Amrit M. Mirajkar, Sumanth B. Shetty

**Affiliations:** 1 Department of Physiology, Nitte (Deemed to be University), Mangalore 575018, India; 2 Department of Psychiatry, Nitte (Deemed to be University), Mangalore 575018, India; 3 Department of Paediatrics, Nitte (Deemed to be University), Mangalore 575018, India

**Keywords:** autonomic nervous system, autism spectrum disorders, heart rate variability, high frequency, low frequency, vanillylmandelic acid

## Abstract

*Background and objectives:* Autism Spectrum Disorder (ASD) is a complex neuro-developmental disorder and it has been suggested that symptoms of ASD are associated with neural networks that regulate the Autonomic Nervous System (ANS). However, the nature of autonomic atypicalities in ASDs remain largely unknown. Measures like Heart Rate Variability (HRV) and urinary Vanillylmandelic Acid (VMA) estimation are sensitive and non-invasive physiological and biochemical indicators of autonomic nervous activity. This study aimed to compare the physiological and biochemical autonomic indices in children with and without ASD. *Materials and Methods:* In this case-control study, 40 children with autism and 40 Typically Developing (TD) children were recruited. Measures of physiological autonomic index were assessed by the analysis of short term HRV, and the urinary levels of VMA estimation was used as a biochemical autonomic index. *Results:* Cardiac sympathetic activity assessed by Low Frequency (nu) of HRV was significantly higher in the ASD group in comparison with the TD group (*p* = 0.006). On the contrary, both the High Frequency (abs) and (nu) of HRV were found to be significantly lower in autistic children (*p* = 0.034 and *p* = 0.000) than controls. Autistic children also exhibited a significantly higher level (*p* = 0.049) of VMA concentration compared to TD children. *Conclusion:* The study concludes that children with ASD exhibit lower cardio-vagal activity as measured by HRV and increased sympathetic activity as assessed by urinary VMA compared to that of TD children. The core autistic symptoms exhibited by children with ASD could be due to the differences in baseline arousal or stress which might be associated with autonomic dysfunction. Further studies are needed to examine the association of this autonomic dysregulation with ASD symptoms and comorbidities.

## 1. Introduction

The physiological responses provided by the peripheral Autonomic Nervous System (ANS) are critical for maintaining homeostasis, physiological flexibility as well as acute adaptations to stressful situations which are mediated through multiple chemical coding systems thus maintaining body homeostasis. The areas of the central autonomic network like brainstem, prefrontal cortex, amygdala, limbic system, and hypothalamus are associated with autonomic control that integrates autonomic function [1]. ANS also is responsible for cognitive, affective, and behavioural responses and its deregulation is found in diverse neuro-psychological disorders. An extensive body of literature suggests that Autism Spectrum Disorders (ASD) symptoms are associated with pervasive atypicalities in the central nervous system, including structures and networks involved in the regulation of the autonomic nervous system [2]. Clinical manifestation of autonomic dysfunction disorders is extremely varied owing to the pervasive effects of ANS and its central autonomic network component on multiple organ systems [1].

ASD is a complex neurological and developmental disorder that impairs ability of a person to communicate and interact with others, displaying significant heterogeneity of symptoms that affects multiple organ systems of the body. The core behavioural symptoms in ASD include atypical social and communicative development along with restricted interests, repetitive behaviours and stereotypical activities. Even though the exact etiology of autism still remains unclear, evidences from recent studies clearly points to the fact that ASD is not a single clinical entity but a behavioural display of tens or hundreds of genetic disorders, which may also be influenced by environmental factors [3]. The behavioural traits exhibited in autistic children like mood instability, hyperactivity, inattention, resistance to change, sensory disintegration, and symptoms attributable to other organ systems including sleep disorders, urinary retention, and gastrointestinal dysfunction can also be attributed to symptoms of autonomic dysfunction exhibited in multiple organ systems [4]. Even though the diagnosis of ASD is mainly based on behavioral assessment, various physiological measures are also been used to determine the neurological or autonomic dysfunctions underlying ASD [5]. The mutual regions of the brain that are associated with both autonomic dysfunction and socio-emotional deregulations, make autonomic status a good biomarker for ASD.

Despite this early evidence of ANS dysregulation in ASD, a wide variability in samples, methods, and measures has produced inconsistent findings. Recently, research on ASD has been focused mainly on ANS activity, due to its potential role in regulating emotional and behavioural functions [6] and several authors have studied autonomic function in children with ASD, by the assessment of autonomic cardiac control. As suggested by literature, the link between ASD symptoms and ANS dysfunction can be related to parasympathetic underactivity, sympathetic over-arousal, or an atypical interaction between these systems [6,7,8]. Some studies have reported no significant differences in resting autonomic activity in children with ASD compared to controls [9,10]. Both sympathetic and parasympathetic lower resting activity were revealed in studies done by Bujnakova et al. in 2016, indicating autonomic under arousal in ASD children [3]. Studies assessing orthostatic stress in ASD children have shown higher parasympathetic responses with the same sympathetic modulation, suggesting parasympathetic dominance in this population [11]. Thus, the inconsistencies in the existing literature on autonomic function in ASD propose a large heterogeneity in this population.

Heart Rate Variability (HRV), a marker of autonomic cardiovascular function, is considered a reliable method for testing ANS functionality. It provides the instantaneous variation in heart rhythm due to autonomic influence on the sinoatrial (SA) node [11]. It is a useful non-invasive tool to study central processes involved in autonomic regulation, thereby emphasizing its relevance in various psychiatric conditions. As per literature, fluctuations in HRV reflects both the sympathetic and parasympathetic responses and the sympathovagal balance can also be assessed [12]. Various HRV indices are widely recognized as useful and powerful indicators of physiological and psychological interaction [13]. At rest, HRV remains large and becomes more regular when influenced by stressful environmental factors. Lower HRV indicates reduced parasympathetic cardiac control and has been associated with disorders ranging from Type 2 diabetes mellitus to sleep problems, anxiety, and emotional disorders in children [14]. In various populations with cardiovascular pathologies, reduced HRV is also recognised as predictor for a higher mortality or cardiovascular events [15].

Neurotransmitters are considered to play a key role in the normal development of brain, memory, motor activity, and behaviour regulation. Henceforth, neurotransmitter system dysfunction affecting neuronal cell migration, differentiation, synaptogenesis and ultimately developmental processes of the brain, is also suggested as a cause of ASD [16]. Estimating the plasma or urine concentration of norepinephrine, or its metabolites, can be used to assess sympathetic activity [17]. The metabolism of catecholamines occurs via multiple pathways resulting in the production of a wide variety of end metabolites, Vanillyl Mandelic Acid (VMA) being one among them. VMA being excreted in urine in large amounts, makes its measurement simple, easy to implement, and is therefore widely used in diagnosis [18]. Plasma norepinephrine concentrations have been reported to be elevated in autism in studies done by Launay et al. (1987) [17]. Reduced urinary free catecholamine concentration was reported in a group of boys with childhood autism compared to normal boys of similar age by Young et al. in 1978 [19]. Increased concentration of urinary homovanillic acid which is a degradation product of dopamine is also reported in patients with ASD [20]. However, to the extent of our knowledge, no study has been performed till date assessing both HRV and urinary VMA concentrations in ASD children as an autonomic index.

Based on all these studies, we addressed the hypothesis that ASD children are characterized by altered autonomic activity. Therefore, this study aimed to compare the physiological and biochemical autonomic indices in children with and without ASD by assessing short term HRV and urinary VMA concentration.

## 2. Materials and Methods

This is a case-control study conducted during the period from April 2016–September 2018, in K. S. Hegde Charitable Hospital, Deralakatte and Special schools in and around Mangalore, Karnataka, India.

### 2.1. Study Population

**Figure 1:**
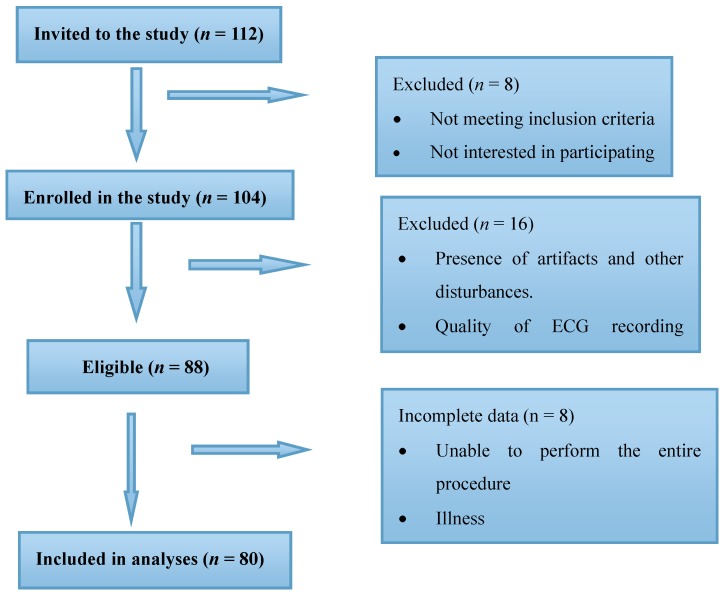
Flow chart of study sample recruitment.

Two groups of children within the age group of 3–14 years participated in this study; (a) children diagnosed with ASDs (*n* = 40) and age and gender matched (b) Typically Developing (TD) children (*n* = 40). A total of 112 children were recruited for the study including 63 autistic children and 49 TD children. However, reliable statistical data could only be obtained for 40 autistic (*n* = 40) and 40 TD (*n* = 40) children. Out of the autistic children 24 were boys and the rest (16) were girls, whereas the TD group comprised of 26 boys and 14 girls. Details of the sample recruitment is provided in Figure 1.

### 2.2. Study Setting

Subject recruitment and ECG recording for HRV analysis were conducted in K. S. Hegde Charitable Hospital, Deralakatte, and Special schools in and around Mangalore. Data analysis was performed in the research lab of the Department of Physiology of K. S. Hegde Medical Academy, Mangalore. Autistic children were recruited from Psychiatric, Paediatric and Speech-language pathology departments of K.S Hegde Charitable Hospital, Special schools, and speech therapy centres in Mangalore. TD children were recruited from nearby schools and had no history of mental and neurodevelopmental disorders and had normal school performance. ASD children and TD children were all investigated under the same conditions.

### 2.3. Ethical Consideration

The study was conducted according to the guidelines of the Declaration of Helsinki, and all procedures involving human subjects were approved by the Central Ethics Committee of Nitte University and the Institutional Ethics committee of K. S. Hegde Medical Academy, Deralakatte, Mangalore. The ethical approval number is NU/CEC/2017-2018/0104, accessed on 13/02/2017. Each subject and his/her parents received written and oral information about the nature and purpose of the study and presented a signed informed consent form before participating in the study.

### 2.4. Screening of Study Subjects

The diagnosis of ASD was made by an experienced Psychiatrist or Clinical Psychologist according to the Diagnostic and Statistical Manual of Mental Disorders (DSM-5) criteria [21] and based on previous psychiatric reports. A medical practitioner confirmed the ASD diagnosis in the autistic group and normal functioning in the control group. The severity of autism was rated using the Childhood Autism Rating Scale (CARS-2), which is a behaviour rating scale. It consists of two 15-item rating scales which are completed by a trained clinician, and a Parent/Caregiver questionnaire. CARS-2 identifies children 2 years and older with ASD and distinguishes between mild to moderate and severe autism.

### 2.5. Inclusion and Exclusion Criteria

Inclusion criteria used for enrolling children for the ASD group were: Primary ASD diagnosis, ability to perform the study, absence of any psychiatric symptoms and other comorbid mental disorders, and this was confirmed by a supervised specialist in child and adolescent psychiatry according to DSM-5 diagnostic criteria prior to examination on the same day. Selected children had neither cardiac nor respiratory diseases that may alter the HR response. None of the participants in either group were prescribed any psychiatric medications nor had documented comorbid disorders (e.g., attention-deficit/hyperactivity disorder, obsessive-compulsive disorder, seizure disorder) at the time of the study. Children with acute infections and diagnosis of disruptive behaviour disorders, profound Intellectual disability (IQ < 70), with disorders of autonomic dysfunction and any other medical illness at the time of recording were excluded as all these conditions would affect the HRV parameters evaluated in the study.

### 2.6. Data Collection

Subjects satisfying our study criteria were screened by a trained psychologist or paediatrician. Following this, subject anthropometric characteristics like height (m), weight (kg), basal Blood Pressure (BP) and Heart Rate at rest (HR rest in bpm) was recorded during rest. The measurement was conducted on the basis of widely accepted and applied rules, while the height was measured with an accuracy of 0.5 cm and the weight with an accuracy of 0.1 kg. Following this, physiological autonomic measure was evaluated by assessing HRV which is derived from lead II ECG recording. Further, urinary VMA concentration was also estimated as the biochemical autonomic indicator.

#### 2.6.1. Preparation for the Recording

Participants were seated in a comfortable chair in a sound attenuated room with the temperature controlled between 23°C and 25°C. They were allowed to rest initially for 10 minutes to get accustomed to the study setting. Meanwhile, the ECG electrodes were placed and the equipment setting and the quality of records were tested. Only the recording person, speech therapist, and parent/caregiver of the subject were present during the study procedure. A familiar person accompanied the participants to increase comfort with the setting and experimental phases. The time required for recording varied from subject to subject depending on their psychological status during the procedure. For TD children the study procedure went on for approximately 20–25 minutes, including the initial time taken for subjects to adapt to the study setting.

#### 2.6.2. Assessment of Physiological Autonomic Activity

The cardiac autonomic activity was assessed using HRV indices, derived from lead II ECG. For recording ECG, firstly, discoid Ag/AgCl electrodes were placed on the subject’s skin after thorough cleaning. These are the most common electrodes used for recording biological signals as it generates low noise level during the recording. Lead II Electrocardiogram (ECG) was then recorded using a computerised 4-channel data acquisition unit (Power lab 26-T, AD instruments, New South Wales, Australia) in sitting position for 5 minutes.

#### 2.6.3. Analysis of HRV Indices

From the ECG recording, a sequential series of successive R-R intervals were obtained which was validated before analysis using a standardised procedure as recommended by the Task Force of European Society of Cardiology [12]. From the raw signal obtained, all possible artifactual beats were eliminated up to a maximum of 2% which could be ectopic beats, arrhythmic events, missing data, and electrical ‘noise’. Finally, a series of rectified R–R intervals were obtained. The data so gathered were then subjected to short term spectral analysis of HRV using Fast Fourier Transform (FFT). Frequency domain analysis of HRV indices were calculated on the most stable 256 consecutives R-R intervals which quantified the spectral density of the Low Frequency (LF), 0.04 to 0.15 Hz and the High Frequency (HF), 0.15 to 0.40 Hz power bands and the total power (TP), variance of all RR intervals all expressed in ms^2^ (Task Force1996). LF components represent both parasympathetic and sympathetic modulations [22] whereas HF is associated to parasympathetic modulation [12]. LF/HF ratio was also calculated, which constitutes evaluation of the ANS balance (sympathetic/parasympathetic). If this ratio is <1, it shows a parasympathetic predominance, whereas a ratio above 1 reflects sympathetic predominance [8,12]. From the report obtained, total power (TP in ms^2^), absolute, and normalized units of HF and LF - HRV indices and LF/HF ratio were considered for statistical analysis.

#### 2.6.4. Estimation of Urinary VMA Concentrations

Proper guidelines for the collection of the urine were explained to the subject. Subjects were asked to avoid the consumption of substances like banana, vanilla-containing food, coffee, and chocolate that can interfere with VMA estimation before urine collection [18]. Approximately 10 ml of 24 h urine sample was collected from all subjects in sterile urine containers and stored at −80 °C to maintain the viability of the metabolites present. VMA analysis was later performed using suitable ELISA kits and the results were analysed.

### 2.7. Statistical Analysis

For each quantitative index in this study, the Gaussian/non-Gaussian distribution was ascertained by applying the Shapiro–Wilk normality test and accepted if the results were *p* > 0.05. For the parametric distribution the mean accompanied by Standard Deviation (SD) values were presented and Student’s t-test was applied to compare indices between ASD and TD groups, while for the nonparametric distribution the median accompanied by minimum and maximum values (IQ range) were presented and the Mann–Whitney U test was applied for comparison. Probability value *p* < 0.05 was considered as statistically significant. All the statistical analysis was performed using the SPSS 20.0. (SPSS- Inc., 233 South Wacker Drive, Chicago, IL, USA) Software package.

## 3. Results

### Subject Characteristics

The general subject characteristics are summarized in Table 1. The results suggest that autistic children exhibited a significantly higher BMI kg/m^2^ compared to their non-autistic counterparts with a *p*-value of 0.01. All other characteristics like age, height, weight, SBP, DBP, and basal heart rate did not show any significant difference between the groups. SBP, DBP, and heart rate were found to be higher in the autistic group but did not reach a statistically significant level.

**Figure 2:**
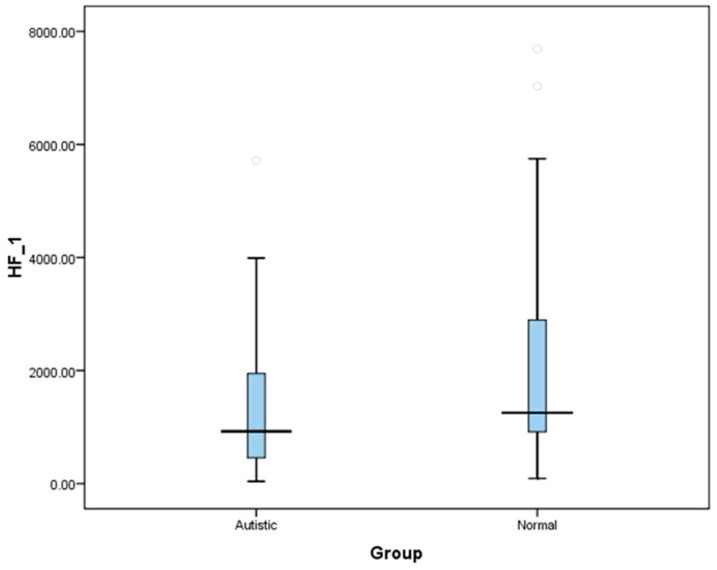
Graphical representation depicting statistical significance of HF absolute values between ASD and TD children. Statistical significance (*p* < 0.05); Abbreviations: HF_1 = high frequency index in absolute values.

**Figure 3:**
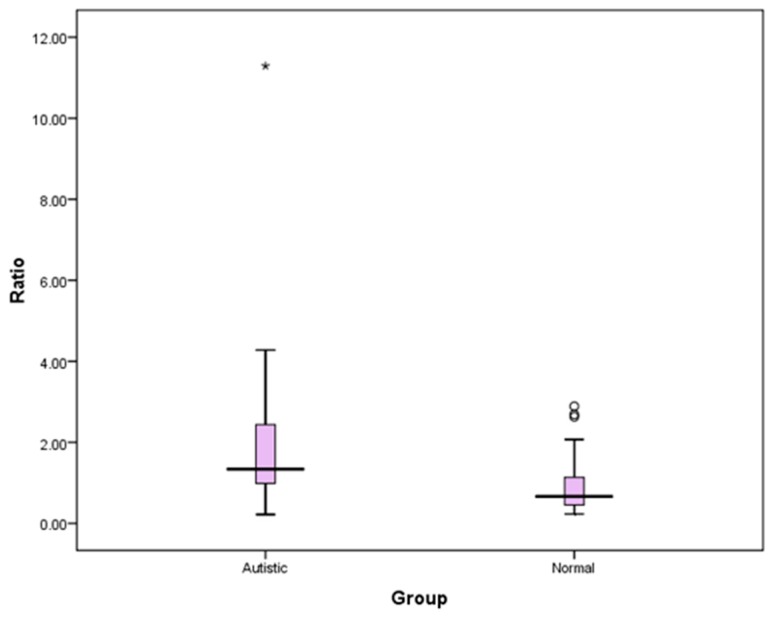
Graphical representation depicting statistical significance of LF/HF ratio between ASD and TD children. * Statistical significance (*p* < 0.05); Abbreviations: Ratio = LF/HF ratio between groups.

Table 2 shows comparison of HRV indices between the study groups. The HF absolute units were found to be significantly lower in the ASD group than the TD group (*p* = 0.034); on the contrary, the LF absolute indices, despite exhibiting a higher range in ASD, was not significantly higher (*p* = 0.381) compared to their non-autistic counterparts. Figure 2 depicts the significantly lower HF (abs) units value in ASD compared to TD children. The HF and LF nu followed a normal distribution and showed a statistical significance between the groups. HF (nu) was significantly lower in the ASD group (*p* = 0.000), whereas LF (nu) was significantly higher in the ASD group in comparison with their TD group *(p* = 0.006). Owing to the higher LF and Lower HF (nu) values in the ASD group, their ratio also revealed a significantly higher value (*p* = 0.000) between the groups. Figure 3 is the graphical representation of statistical significance of the LF/HF Ratio between the groups. The TP values (*p* = 0.870) on the other side did not exhibit any significant difference between the groups.

**Figure 4:**
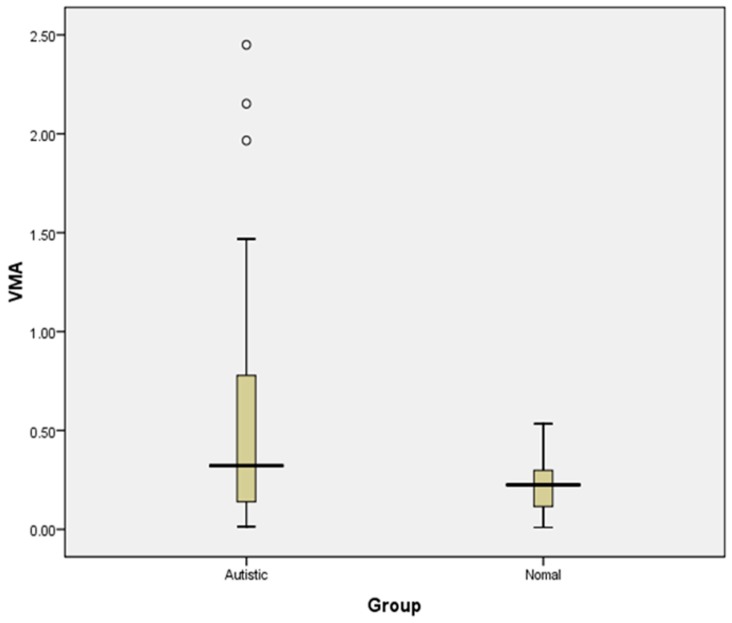
Graphical representation depicting statistical significance of VMA concentration between ASD and TD children. * Statistical significance (*p* < 0.05); Abbreviations: VMA—VanillylmandelicAcid concentration in mg/24 h.

Table 3 and Figure 4 shows that the autistic children exhibited a significantly higher level (*p* = 0.044) of VMA concentration compared to TD children, but still within the normal range. The normal reference range for urinary VMA level in Children 3–8 years < 2.3 mg/24 h, Children 9–12 years < 3.4 mg/24 h and Children 13–17 years <3.9 mg/24 h. Table 4 depicts the correlation of HRV and urinary VMA concentration with severity of autism. No significant correlation was found between severity of autism and any of the indices.

## 4. Discussion

ASD symptoms are associated with pervasive abnormalities in the central nervous system including structures and networks involved in the regulation of ANS. Physiological measures like HRV and biochemical indices like urinary VMA estimation offer sensitive and non-invasive indicators of autonomic nervous activity. Although many studies have reported alterations in ANS activity in adults and children, not many data are available on cardiac and biochemical autonomic measures in children with ASD. The current study compared the cardiac and biochemical autonomic patterns between ASD and TD children by assessing HRV and urinary VMA, respectively. Findings of our study showed that the ASD population exhibits lower cardiac vagal activity as assessed by HRV and increased sympathetic measure as assessed by urinary VMA compared to that of TD children.

Analysis of anthropometric measures showed that the ASD group had higher BMI than TD children, indicating a greater risk of developing chronic diseases such as heart disease and diabetes among the ASD group in their later life. Higher BMI observed among the ASD group might be due to their sedentary lifestyle, stereotypical and unhealthy feeding habits, and rarely due to underlying genetic causes.

The results of cardiac autonomic measures showed that ASD children are associated with reduced baseline cardiac parasympathetic, increased sympathetic activity, and therefore elevated sympathovagal balance. This is reflected by lower HRV indices like HF-HRV (abs and nu) and higher HRV indices in LF-HRV (nu) and LF/HF ratio among ASDs. Similar results were obtained on the comparison of these variables between ASD and TD children in the category of two age groups. Further, our study population recruited in ASD and TD groups were age-and gender matched, indicating that the covariates like age and sex did not contribute for the differences in autonomic indices observed between our study groups.

Measures of HRV are being increasingly applied in investigations of the central autonomic state and to study the fundamental links between various psychological processes and physiological functions [13]. As the HF-HRV spectrum assesses parasympathetic activity, the difference in parasympathetic response in our study suggests that children with ASD are more stressed under ‘resting’ conditions than TD children. LF spectrum is a parameter that includes both sympathetic and parasympathetic activities [22,23] and the presence of a significant difference of LF values, and LF/HF ratio between our two groups confirms that ASD and TD groups have an altered cardiac autonomic function. Our findings of decreased cardiovagal modulations as manifested by decreased HF-HRV components are consistent with most of the previous studies reporting decreased vagal cardiac regulation in ASD [6,7,8,24]. Studies in children with ASD compared to TD controls under resting conditions as well as during mental stress have frequently reported increased heart rate suggesting an increased sympathetic activity [2,6]. Autistic children exhibited an increased sympathetic activity, whereas TD children exhibited a decrease in sympathetic influence during social interaction with a familiar person [24]. Studies by Porges et al. in 2003 have revealed that autonomic imbalance in the form of sympathetic over activity and low parasympathetic activity can be associated with difficulties in social behaviour resulting in a potentially higher risk of cardiovascular morbidity in ASD children [25]. An elevated LF\HF ratio and reduced HF-HRV indices thoroughly replicates a dominant sympathetic balance, in which the “fight or flight” response is hyperactive, and the parasympathetic “brakes” on the autonomic nervous system are under-utilized [26]. The recent conceptualizations of stability and instability in biological systems depends mainly on autonomic flexibility displayed within the body in response to changing environmental conditions, which autistic children are lacking [27]. Thus, the stereotypical behavioral response in ASD could be associated with their impaired autonomic nervous activity.

A comparison of urinary VMA levels between two study groups reveals an increase in urinary VMA concentration in the ASD group compared to the TD group, indicating increased sympathetic activity in the ASD group. Studies on serotonin and catecholamine neurotransmitter systems strongly suggest that neurochemical factors play a major role in autism [28]. In the present study, urinary VMA, a major metabolite marker of catecholamine, was significantly higher in autistic children than in the control group, which may be related to frequent stressful situations to which autistic children are subjected.

Even though the estimated urinary VMA values fall in the normal range for both the groups, there is a trend of increased levels of urinary VMA concentration in the ASD group compared to controls, though not statistically significant. The outcome of urinary VMA analysis supports the finding of cardiac sympathetic overactivity, as obtained from HRV analysis among the autistic group compared to controls. Further studies with a higher sample size may be able to conclude whether it can be used as a biomarker for autonomic dysfunction ASD.

Aspects like worse handling of stress, over-elicitation of the physiological response, or dysfunctional stress response systems might contribute to the increased response of autistic children to stressors [29]. However, due to the pervasive effects of ANS on multiple systems, clinical manifestations can vary widely, not only between various disorders but also within a particular disorder [4]. Though the measurement of salivary cortisol is a biomarker for the evaluation of acute stress, our study did not involve any task/activities inducing acute stress, and all the autonomic parameters were assessed under basal/resting conditions. Therefore, we did not consider the evaluation of salivary cortisol levels in our study population. However, the measurement of hair cortisol would have provided the levels of chronic stress which was not performed in our study population and that is one of the limitations of this study.

Autism severity of the study subjects were evaluated by administering the Childhood Autism Rating Scale (CARS). Based on the obtained scores, we assessed the strength of association of severity of autism with cardiac autonomic and biochemical parameters using Spearman rank correlation for non-parametric variables and Pearson correlation for parametric variables. However, there was no statistically significant correlation of severity with any of the variables (Table 4). This could be due to the relatively lesser number of autistic children in the severe grade in our study.

Our study was the first of its kind in an Indian Scenario to investigate both physiological and biochemical autonomic measures in the same study population of ASDs. Our finding is based on a relatively small sample size; therefore, evaluation including a larger study population and using sensitive psycho-physiological measures like sympathetic skin response would help in the better understanding of autonomic neural activity in autism spectrum disorders. The study of autonomic regulation in childhood psychiatric disorders may provide a better understanding of the etiology and aids in the prevention of cardiovascular diseases in adults [30]. Therapeutic interventions in autonomic dysfunctions and monitoring of autonomic functions will provide a new avenue for both the understanding and treatment of autism. Aggression, anxiety, as well as the core symptoms of autism can be treated through interventions focused on autonomic function [5]. The finding of ANS dysregulation in this study may ultimately suggest new treatment targets in ASD, and lead to inexpensive and non-invasive markers for deficits associated with this disorder.

## 5. Conclusions

The findings of our study showed that children with ASD exhibit lower cardiac vagal activity as measured by short term HRV and increased sympathetic activity assessed by urinary VMA compared to that of TD children. The core autistic symptoms which include impaired social interaction, repetitive and stereotypical behaviour could be a result of the differences in baseline arousal or stress which may be associated with impaired autonomic nervous activity. Future studies are needed to examine the association of this deregulation of ANS with symptoms and co-morbidity of ASDs.

## Figures and Tables

**Table 1:** Subject characteristics.

Characteristics	ASD (*n* = 40)	TD (*n* = 40)	*p*-Value
Age (years)	10 (5.25, 12)	9 (7.25, 11.75)	0.87
Weight (kg)	23.5 (19.25, 39.5)	24.5 (19, 31)	0.75
Height (m)	1.28 ± 0.22	1.32 ± 0.16	0.52
BMI (kg/m^2^)	16.76 (13.71, 18.94)	14.35 (13.38, 15.44)	0.01 *
SBP (mm Hg)	112.6 ± 8.97	110 ± 6.19	0.19
DBP (mm Hg)	76.92 ± 5.39	75.67 ± 4.1	0.25
Basal Heart rate (bpm)	82 (78, 89.8)	79 (74.25, 86)	0.08

* Statistical significance (*p* < 0.05). Descriptives: Mean ± SD, Median (25th–75th percentile); Abbreviations: BMI—Body Mass Index, calculated as Weight (kg)/Height (m^2^)**,** SBP—Systolic Blood Pressure, DBP—Diastolic Blood Pressure.

**Table 2:** Physiological indices.

HRV INDICES	ASD (*n* = 40)	TD (*n* = 40)
**HF absolute (ms^2^)**	924.58 (456.12–2031.06) *	1253.3 (897.66 –3182.22)
**HF normalized (nu)**	33.69 ± 13.22 *	48.31 ± 14.64
**LF absolute (ms^2^)**	1182.89 (703.18–2040.12)	1108.38 (558.39–1943.44)
**LF normalized (nu)**	48.19 ± 16.2 *	38.88 ± 13.3
**TP (ms^2^)**	3415.69 (2670.23–6098.48)	3938.19 (2463.84–6868.43)
**LF/HF ratio**	1.34 (0.98–2.47) *	0.67(0.45–1.15)

Statistical significance (*p* < 0.05). Descriptives: Mean ± SD, Median (25th–75th percentile; Abbreviations: (HF—High frequency, LF—Low frequency, TP—Total power), * *p* < 0.05.

**Table 3:** Urinary VMA concentration.

Biochemical Index	ASD (*n* = 40)	Control (*n* = 40)	*p*-Value
VMA levels (mg/24 hrs)	0.32 (0.14, 0.8)	0.23 (0.12, 0.31)	0.044 *

VMA—VanillylmandelicAcid, * *p* ≤ 0.05.

**Table 4:** Correlation of autonomic indices with severity of ASD.

Autonomic Indices	ASD Severity	
	R	*p*-Value
**HF absolute (ms^2^)**	0.034	0.83
**HF normalized (nu)**	0.06	0.71
**LF absolute (ms^2^)**	0.17	0.29
**LF normalized (nu)**	0.08	0.63
**TP (ms^2^)**	0.04	0.81
**LF/HF ratio**	0.02	0.89
**VMA levels (mg/24 h)**	0.33	0.05

Statistical significance (*p* < 0.05); Abbreviations: HF—High frequency, LF—Low frequency, TP—Total power, VMA—Vanillylmandelic acid).
